# Metabolic heterogeneity of follicular amino acids in polycystic ovary syndrome is affected by obesity and related to pregnancy outcome

**DOI:** 10.1186/1471-2393-14-11

**Published:** 2014-01-10

**Authors:** Chun-mei Zhang, Yue Zhao, Rong Li, Yang Yu, Li-ying Yan, Li Li, Na-na Liu, Ping Liu, Jie Qiao

**Affiliations:** 1 Reproductive Medical Center, Department of Obstetrics and Gynecology, Peking University Third Hospital, No. 49 Huayuan North Road, Beijing, Haidian District, 100191, People’s Republic of China; 2 Key Laboratory of Assisted Reproduction, Ministry of Education, Beijing, China; 3 Beijing Key Laboratory of Reproductive Endocrinology and Assisted Reproductive Technology, Beijing, China

**Keywords:** PCOS, Amino acid, Obesity, Follicular fluid

## Abstract

**Background:**

Polycystic ovary syndrome (PCOS) is a heterogeneous endocrine disorder frequently accompanied by obesity and by insulin resistance, and patients with this syndrome suffer from infertility and poor pregnancy outcome. Disturbances in plasma amino acid (AA) metabolism have been implicated in women with PCOS. However, direct evidence on follicular AA metabolic profiles in PCOS patients and their relationship with pregnancy outcome is sparse.

**Methods:**

We conducted a prospective study in 63 PCOS patients and 48 controls in the Division of Reproductive Center, Peking University Third Hospital. Follicular AA levels were measured by the liquid chromatography-tandem mass spectrometric method, and the results were analyzed based on different grouping criteria.

**Results:**

The levels of aromatic amino acid (AAA) increased in PCOS patients independent of obesity (*P* < 0.05), whereas the levels of branched-chain amino acid (BCAA), glutamic acid, phenylalanine, alanine, and arginine increased with body mass index irrespective of the PCOS status (all *P* < 0.05). In addition, compared with non insulin resistant-PCOS patients and controls, insulin resistant-PCOS group had higher levels of leucine, valine and glutamic acid (all *P* < 0.05). In PCOS group, aspartic acid and serine levels were elevated in pregnant patients compared with the non-pregnant subjects (both *P* < 0.05). Moreover, the levels of BCAA and valine were higher in the non-pregnant group than in the pregnant group (both *P* < 0.05). The pregnancy rate (45.00%) of subjects with elevated BCAA level was significantly lower than that (66.67%) in control subjects (*P* = 0.036) at a BCAA cutoff value of 239.10 μM, while the abortion rate was much higher (33.33% versus 2.78%, *P* = 0.004).

**Conclusions:**

Both PCOS and obesity were accompanied by follicular AA metabolic disturbances, with obesity exerting a more pronounced effect on AA metabolic profiles. The disruptions in specific AAs in the follicular fluid might account for the inferior pregnancy outcome in obese patients and increased risk of abortion in PCOS patients.

## Background

Polycystic ovary syndrome (PCOS) is a complex and heterogeneous endocrine disorder that is associated with multiple metabolic abnormalities [1] including obesity and insulin resistance (IR). Epidemic studies demonstrate that women with this syndrome have an increased risk of developing type 2 diabetes mellitus (T2DM) and cardiovascular diseases [2]. Besides these long-term complications, infertility and poor pregnancy outcome are the major problems in PCOS patients.

As the most common cause for anovulatory infertility, PCOS affects 5–10% of reproductive-aged women [3]. Although significant progress has been made in the treatment of PCOS, a few challenges still remain. For example, more oocytes could be obtained from PCOS patients undergoing *in vitro* fertilization (IVF), but they are often of poor quality with higher miscarriage rate [4], indicating systemic or local endocrine/metabolic disruptions may alter the intrafollicular microenvironment during folliculogenesis. Thus, for a better understanding of the molecular mechanisms of pregnancy-related dysfunction, further studies are needed to uncover the metabolites favorable for oogenesis and better pregnancy outcome in PCOS women.

In a recent metabonomic study, we observed abnormal changes of various metabolites in the plasma of PCOS women, among which the change of amino acids (AAs) metabolic profile was especially remarkable and related to IR, obesity and anovulation [5]. Aside from their critical roles in supplying calories, various AAs serve as regulatory signals with hormone-like functions and are implicated in IR, inflammation and embryo implantation [6-8], indicative of a close relationship between abnormal AA metabolism and PCOS pathophysiology.

The studies on the metabolic profiles of PCOS patients so far are restricted to the plasma level [5,9,10]. However, systemic metabolic disturbances may be reflected in the local ovarian environment, i.e., follicular fluid (FF) that contains metabolites crucial for oocyte growth and reflective of embryo viability and oocyte quality [11]. In addition, data on the relationship between AA metabolism and pregnancy outcome in PCOS patients undergoing IVF-embryo transfer (IVF-ET) treatments are not yet available. Based on these previous findings, we hypothesized that disturbances of AA might also be present in the FF of the patients, which provide an adverse microenvironment and negatively influence oocyte quality, embryo development and pregnancy outcome. In the current study, we measured the levels of 20 natural AAs in the FF in PCOS and control women, and analyzed the data based on structured grouping criteria. Our study may help unravel the metabolic disturbances in PCOS patients and provide valuable directions to clinical treatments.

## Methods

### Study populations

This study was approved by the Ethics Committee of Peking University Third Hospital. Informed consents were obtained from all women prior to inclusion in this study.

Subjects included 63 PCOS patients and 48 control women who visited the Division of Reproductive Center, Peking University Third Hospital from February to October in 2012. PCOS was diagnosed according to the 2003 Rotterdam criteria [12], i.e. the presence of two of the following three criteria: oligo- or an-ovulation, signs of clinical hyperandrogenism and/or biochemical signs of hyperandrogenism and polycystic ovaries on ultrasonography after exclusion of other aetiologies. The control group included women attending the clinic on account of male azoospermia or tubal occlusion. Women exposed to any hormonal treatment or insulin-lowering agent during the last 3 months were excluded from the study.

Patients received a standard gonadotropin releasing hormone (GnRH) agonist (diphereline) regimen starting on day 21 of a spontaneous menstrual cycle. Follicle-stimulating hormone (FSH) stimulation was initiated once down-regulation was confirmed via ultrasound and serum estradiol (E2) measurement. HCG (10000 IU) was administered when at least three follicles reached 18 mm in diameter. Oocyte retrieval was performed 36 h later under transvaginal ultrasound guidance. All patients received luteal phase support using vaginally administered progesterone starting from the day after oocyte retrieval. Embryos or blastocysts were transferred on the third or the fifth day after oocyte retrieval. Depending on the age of the subject and embryo quality, one to three embryos were transferred. Clinical pregnancy was defined as the presence of a gestational sac on ultrasound performed at 6 weeks after embryo transfer.

### Sample preparation and laboratory assays

Fasting blood samples from all subjects were collected on days 2–5 of a natural cycle or when amenorrhea for over 40 days with follicle diameter not exceeding 10 mm for basal FSH, luteinizing hormone (LH), androstenedione (A), and E2 assay. Fasting glucose and insulin levels were measured within 2 h after blood sampling on the day of oocyte retrieval. FF was aspirated from the leading follicle from each ovary. Only FF macroscopically free from blood was retained for further determinations. FF samples were centrifuged for 10 min at 3000 g and then stored at -80°C until use.

AA concentrations were determined by the liquid chromatography-tandem mass spectrometric method using a 3200 Q TRAP triple-quadrupole linear ion trap mass spectrometer (Applied Biosystems, Darmstadt, Germany) and a high performance liquid chromatography system (Dionex, Sunnyvale, CA, USA) as described previously [13]. Sex hormones were determined by chemiluminescence (Siemens, Germany).

### Data analysis

Comparisons of the quantitative and categorical data were made by SPSS 16.0 using two-tailed t test and Chi-square test or Fisher’s exact test, respectively. Multiple linear regression analysis was used to discriminate independent effects of each variable. Pearson correlation test was performed to evaluate the relationship of variables. Values were presented as means ± SEM. *P* < 0.05 was considered statistically significant.

## Results

### Baseline characteristics of control and PCOS subjects

Characteristics of the PCOS and control cohorts were summarized in Table 1. There was no difference in age between the two groups. PCOS patients had higher body mass index (BMI) than the control counterparts (*P* = 0.025). Compared with the controls, PCOS patients exhibited elevated levels of LH and A (*P* < 0.001). In addition, the ratio of LH to FSH was also higher in the PCOS group (*P* < 0.001). Consistent with the finding that PCOS patients are often associated with IR, the fasting insulin level and HOMA-IR were higher than those in the control group (*P* < 0.001). However, the fasting glucose concentrations were similar between the two groups.

**Table 1:** The clinical characteristics of subjects

	**Control (n = 48)**	**PCOS (n = 63)**	***p*-value**
Age (y)	29.88 ±0.51	29.60 ± 0.32	0.653
BMI (kg/m^2^)	22.84 ±0.49	24.40 ± 0.47	0.025
LH/FSH	0.53 ± 0.04	1.30 ±0.08	<.001
FSH (U/L)	6.48 ±0.23	6.02 ±0.22	0.154
LH (U/L)	3.34 ±0.22	7.62 ±0.50	<0.001
A (nmol/L)	7.45 ± 0.51	13.57 ±0.86	<0.001
Fasting glucose (mmol/L)	4.73 ± 0.07	4.72 ± 0.07	0.949
Fasting insulin (μU/L )	7.60 ± 0.49	11.37 ± 0.71	<0.001
HOMA-IR	1.54 ± 0.12	2.42 ± 0.16	<0.001

Values were expressed as mean ± SEM. Comparisons between groups were done with t test after testing for equality of variance. BMI, body mass index; HOMA-IR: homeostatic model index of insulin resistance.

### Follicular AA metabolic characteristics

Follicular AAs levels in the two groups were shown in Additional file 1: Table S1. Valine, glycine, alanine, and glutamine were the most abundant AAs in the FF. Both PCOS and obesity influenced AA metabolism. Compared with BMI-matched controls, overweight and lean PCOS patients showed elevated levels of tyrosine and tryptophan (Additional file 1: Table S1). We also found that phenylalanine and glutamic acid values increased in obese PCOS and control subjects in comparison with their counterparts, indicating a close association with BMI. Linear regression analysis indicated that the levels of aromatic amino acid (AAA, including phenylalanine, tryptophan and tyrosine) were elevated in PCOS group independent of BMI (*P* < 0.05) (Table 2). In comparison, the levels of branched-chain amino acid (BCAA, including isoleucine, leucine and valine), glutamic acid, phenylalanine, alanine, and arginine were up-regulated in patients with higher BMI irrespective of PCOS status (*P* < 0.05). These results indicate that AA metabolic abnormality was present in PCOS and obese patients, which may negatively affect oocyte development and pregnancy outcome.

**Table 2:** Relationship between follicular AAs levels and PCOS or BMI

**AAs**	**Model 1**^**a**^			**Model 2**^**b**^		
	**β**	**95% CI**	***p*-value**	**β**	**95% CI**	***p*-value**
ILE	1.89	-1.50-5.29	0.561	0.48	0.01-0.94	0.045
LEU	2.51	-4.30-9.33	0.467	1.17	0.24-2.10	0.014
VAL	11.87	-0.85-24.59	0.067	3.06	1.32-4.80	<0.001
PHE	7.40	2.46-12.35	0.004	1.30	0.62-1.97	<0.001
TYR	6.90	2.45-11.35	0.003	0.38	-0.22-0.99	0.214
TRP	4.24	1.54-6.94	0.002	<0.001	-0.37-0.37	0.999
GLU	6.06	-0.96-13.09	0.090	2.39	1.43-3.35	<0.001
ALA	-25.79	-55.39-3.81	0.087	4.32	0.27-8.36	0.037
ARG	1.26	-3.04-5.57	0.561	0.82	0.23-1.41	0.007

Regression coefficient (β) shown, from multiple linear regression analysis in the samples controlling for BMI and PCOS status; CI, confidence interval.

a, adjusted for BMI.

b, adjusted for PCOS status.

Considering that obesity often influences the association of PCOS with IR, it is possible that obesity-related AAs may correlate with IR. To test this possibility, patients were divided into insulin resistant (IR) and non insulin resistant (NIR) groups using HOMA-IR, and the cutoff point was set to 2.69 [14]. 26 patients with PCOS were resistant to insulin, while only 4 IR cases were found in the control group (41.27% versus 8.33%, *P* < 0.001). Compared with the NIR-PCOS and controls, IR-PCOS group had elevated levels of leucine, valine and glutamic acid (*P* < 0.05) (Additional file 1: Table S2), indicative of their roles in the development of IR. Alanine level was also higher in the IR PCOS patients compared with NIR-PCOS (*P* < 0.05). Moreover, NIR-PCOS patient displayed higher AAA concentrations than the NIR-controls, confirming elevated AAA levels as key characteristics of PCOS.

### Pregnancy outcome and its relationship with AAs

Compared with the controls, PCOS patients had increased numbers of fertilized embryos (*P* = 0.032) and total available embryos (*P* = 0.017) (Table 3). The numbers of retrieved oocytes and 2PN stage embryos in PCOS group also tended to increase, but the difference between groups failed to achieve statistical significance. 49 out of 63 PCOS patients received embryo transfer (ET), while it was canceled in 11 patients to prevent ovarian hyper-stimulation syndrome (OHSS). Another 2 patients failed to receive any embryo qualified for transfer, and ET was delayed in 1 patient because of unsuitable endometrium. In the control group, only 3 patients canceled ET for the prevention of OHSS. The cycle cancellation rate in the PCOS group was much higher than that in control group (22.22% versus 6.25%, *P* < 0.05). PCOS patients showed a trend to have a higher abortion rate in comparison with the controls, but the difference was not statistically significant (18.52% versus 7.40%, *P* = 0.224).

**Table 3:** Pregnancy-related indexes grouped by PCOS status or BMI

**Indexes**	**PCOS status**		***p*-value**	**BMI**		***p*-value**
	**Control (n = 48)**	**PCOS (n = 63)**		**≤25 kg/m**^**2 **^**(n = 73)**	**>25 kg/m**^**2 **^**(n = 38)**	
No. of oocytes retrieved	15.40 ± 1.23	18.75 ± 1.30	0.064	18.36 ± 1.19	15.26 ± 1.39	0.111
No. of fertilized embryos	10.52 ± 0.98	13.71 ± 1.08	0.032	13.56 ± 0.98	9.97 ± 1.12	0.025
Fertilization rate	0.68 ± 0.03	0.71 ± 0.03	0.441	0.74 ± 0.02	0.61 ± 0.04	0.007
No. of 2PN embryos	9.04 ± 0.89	11.40 ± 0.96	0.074	11.37 ± 0.86	8.47 ± 0.99	0.040
2PN rate	0.57 ± 0.03	0.58 ± 0.03	0.829	0.61 ± 0.03	0.51 ± 0.04	0.029
No. of available embryos	6.56 ± 0.77	9.49 ± 0.93	0.017	8.64 ± 0.81	7.42 ± 1.03	0.364
**Embryo transfer**						
Yes	45	49	N/A	61	33	N/A
No	3	14	N/A	12	5	N/A
Cancellation rate	6.25%	22.22%	0.021	16.44%	13.16%	0.649
**Pregnancy outcome**						
No. of pregnant cases	25	22	N/A	30	17	N/A
No. of failed cases	18	22	N/A	29	11	N/A
No. of pregnancy loss	2	5	N/A	2	5	N/A
Implantation rate	0.38 ± 0.06	0.33 ± 0.06	0.550	0.33 ± 0.05	0.40 ± 0.07	0.345
Pregnancy rate	60.00%	55.10%	0.210	52.46%	62.07%	0.184
Abortion rate	7.40%	18.52%	0.224	6.25%	22.72%	0.107

Values were expressed as mean ± SEM. Comparisons between groups were done with t test after testing for equality of variance or Chi-square test or Fisher’s exact test for proportional data analysis. PN, pronucleus. N/A, not available.

We further analyzed the effects of BMI on pregnancy outcome by considering all patients as a whole. Patients were grouped as either obese (BMI ≥ 25) or with normal-weight (BMI < 25) [15]. We found that overweight patients had less fertilized embryos and 2PN embryos (*P* = 0.025 and *P* = 0.040, respectively) (Table 3) compared with normal body weight counterparts. Additionally, the fertilization rate and 2PN rate also significantly declined in the overweight group (*P* = 0.007 and *P* = 0.029, respectively), while the abortion rate tended to increase (22.72% versus 6.25%, *P* = 0.107).

Further analyses in pregnant patients (PP) and non-pregnant patients (NPP) revealed the potentially favorable or unfavorable roles of AAs. Patients ended with pregnancy loss were classified as failed patients. Compared with unsuccessful PCOS cohorts, pregnant PCOS patients had significantly higher levels of aspartic acid and serine (*P* = 0.009 and *P* = 0.032, respectively) (Additional file 1: Table S3), and the glycine level tended to be elevated (*P* = 0.065). The levels of BCAA were slightly increased but did not reach statistical significance in the failed group. These results suggest that aspartic acid and serine may serve as favorable metabolites for successful pregnancy in PCOS patients.

As shown above, patients with higher BMI had elevated BCAA levels. The association of BCAA with pregnancy outcome was more prominent when considering all the patients as a whole. A total of 94 subjects received ET in the two groups. The levels of all three BCAAs increased in the unsuccessful group, and significant differences were observed in total BCAA and valine levels (*P* = 0.049 and *P* = 0.047, respectively) (Additional file 1: Table S3). The pregnancy rate of subjects with elevated BCAA levels was significantly lower than that of the control subjects (*P* = 0.036) at a BCAA cutoff value of 239.10 μM, while the abortion rate was much higher than the controls (*P* = 0.004) (Table 4).

**Table 4:** Chi-square test of pregnancy outcome grouped by BCAA values in all embryo-transferred subjects

**Pregnancy outcome**	**BCAA ≤ 239.10 (μM) (n = 54)**	**BCAA>239.10 (μM) (n = 40)**	***p*-value**
Pregnancy rate	66.67% (36/54)	45.00% (18/40)	0.036
Abortion rate	2.78% (1/36)	33.33% (6/18)	0.004

Comparisons between groups were made by Chi-square test or Fisher’s exact test for proportional data analysis.

## Discussion

The current study determined follicular AA levels in PCOS and control subjects. We found that AAA levels were increased in PCOS patients independent of obesity, whereas BCAA, glutamic acid, phenylalanine, alanine, and arginine levels were elevated with BMI irrespective of PCOS. Moreover, in PCOS patients, increased leucine, valine and glutamic acid levels were associated with IR, while elevated aspartic acid and serine levels were associated with pregnancy success. Additionally, in non-pregnant subjects, BCAA and valine levels were higher compared to pregnant subjects. Taken together, our data suggest that both PCOS and obesity were accompanied by follicular AA metabolic disturbances, which may influence pregnancy outcome.

To our knowledge, our study was the first to examine follicular AAs metabolic profiles in PCOS patients. Former reports on AA metabolic profiles in PCOS patients are limited to the plasma level and focused on selected, instead of all, natural AAs except for the study by Zhao and colleagues [5,9,10]. In addition, none of these studies assessed the potential influences of AAs on pregnancy outcome, with which both clinicians and PCOS patients are mostly concerned. In comparison, the current study examined 20 natural AAs and specifically focused on local ovarian environment that is more relevant to oocyte development and pregnancy. The findings on the heterogeneity of AAs profiles associated with PCOS, IR and obesity as well as the relationship between AAs and pregnancy outcome may provide valuable guidance to clinical practice. Notably, several beneficial or unfavorable AAs for pregnancy outcome were highlighted in this study. For example, aspartic acid and serine may improve the chance of successful pregnancy in PCOS patients, while elevated BCAA values may increase the risks of abortion and pregnancy failure in patients with higher BMI. These data suggest that novel therapeutic strategies that could increase the levels of beneficial AAs and/or normalize the levels of unfavorable AAs may promote pregnancy success in PCOS and obese patients.

This study showed that, similar to our findings in the plasma [5]. AAA levels were increased in the follicular fluid in PCOS patients. Additionally, both leucine and valine levels were correlated with IR in both serum and follicular fluid. However, our studies also reveal discrepancies in systemic and local AA metabolic profiles in PCOS patients. Plasma levels of BCAA were increased in PCOS patients, whereas follicular BCAA was mainly affected by BMI. As here we selected both obese and normal-weight patients, while only lean control patients were included by Zhao, the current study design could more precisely distinguish the respective influences of PCOS and obesity. Moreover, the relationship between plasma glycine, serine and threonine levels and IR was not noticed in the follicular fluid. In addition, in the current study, most of the PCOS patients receiving IVF treatments were anovulatory or oligo-ovulatory. Therefore, unlike the study in the plasma, we could not compare the difference of AA levels in patients with and without ovulation.

We observed that elevated AAA level was a typical characteristic of PCOS. However, no difference in AAA levels between pregnant and non-pregnant PCOS subjects was detected, indicating that AAA may not influence pregnancy outcome. Moreover, IR-PCOS subjects showed distinct AA metabolic profiles compared with NIR-PCOS and control subjects. These findings suggest that PCOS patients exhibited heterogeneous follicular AAs profiles.

We noticed that obesity exerted a more pronounced effect on pregnancy outcome and AA metabolic profiles than expected. In agreement with previous findings that obesity is associated with an impaired response to fertility treatments and negatively affects pregnancy outcome [16,17], we found that overweight patients had less fertilized and 2PN embryos as well as reduced fertilization and 2PN rates, which may be attributed to the metabolic disturbances of obesity-related AAs such as BCAA and glutamic acid. Since obesity is the major feature of PCOS, these AAs with various molecular and cellular functions may exert significant effects in PCOS patients and merit further attention. The possible mechanisms of action of the relevant AAs will be discussed below.

We found that the above-mentioned AAs, especially BCAA, were closely related to pregnancy outcome. In obese subjects, the increased levels of glutamic acid, which is produced in the first step of BCAA catabolism, might simply be reflective of elevated BCAA concentrations. Recently, it has been shown that the alterations of plasma BCAA and Glx (mostly glutamic acid) levels are strongly associated with IR [18]. Prospective studies have also verified that BCAA is the predicator of IR and diabetes [6]. The increase in follicular BCAA concentrations in obese and PCOS patients, especially those with IR, may reflect local ovarian IR as their utilization required normal insulin signaling [9]. On the other hand, these elevated AAs may in turn promote the occurrence of IR.

The molecular mechanisms of the action of BCAA involve multiple signaling molecules, especially the insulin receptor substrate-1 [19,20]. Moreover, IR is a key feature associated with PCOS and contributes to reproductive dysfunction in PCOS patients [21]. In addition to the peripheral IR, both granulosa-lutein cells and cumulus cells in PCOS patients display blunted insulin-stimulated glucose uptake and lactate production due to abnormal insulin signaling [22,23], which may further compromise energy supply and cell growth. Taken together, our findings suggest that the elevation of follicular BCAA negatively influences pregnancy outcome possibly via inducing local IR in the ovary. Future studies are needed to dissect their molecular and cellular mechanisms in PCOS and obesity.

Aspartic acid is positively associated with the quality of metaphase II oocytes and fertilization rates in IVF patients [24]. Moreover, aspartic acid supplement can improve semen quality and modulate steroidogenesis [25,26], indicative of its essential role in male reproduction. We found that aspartic acid was markedly elevated in pregnant PCOS patients, and our data suggest it as a beneficial metabolite for pregnancy outcome in PCOS patients. However, we did not observe similar results when all patients were considered as a whole. Therefore, the role of aspartic acid in these processes and whether it exerts differential effects in PCOS and healthy women merit further investigations.

Serine and glycine are another two AAs that attract our attention. The elevated levels of serine in the pregnant PCOS group imply its potentially favorable role in pregnancy outcome. Intriguingly, we found that serine and glycine concentrations were highly correlated in PCOS patients (r = 0.67) (Additional file 1: Figure S1). As reported previously, chronic inflammation, the imbalance between pro- and anti-inflammatory cytokines and impaired glucose tolerance in the plasma and FF are involved in the pathophysiology of PCOS [27,28]. It has also been shown that glycine can increase the levels of anti-inflammatory cytokines while reduce the levels of inflammatory cytokines [7,29,30], which may disrupt steroidogenesis, follicular maturation and ovulation and thus contribute to ovarian dysfunction [31,32]. Moreover, glycine treatment decreases the levels of pro-inflammatory cytokines and improves glucose metabolism in T2DM [33,34]. Therefore, glycine may be considered beneficial for patients with inflammation and impaired glucose regulation, such as PCOS subjects. As for serine, it is possible that it exerts similar effects to glycine via conversion to glycine [35]. The decreased level of follicular serine in PCOS patients may increase the production of local inflammatory cytokines, which consequently causes ovarian dysfunction. However, the involvement of serine in reproduction merits further studies. It should be noted that, similar to the results for aspartic acid, we failed to detect any beneficial effect of serine for successful pregnancy when all patients were considered as a whole, indicating that its role may be limited to PCOS patients.

Our study has a few limitations. First, PCOS patients did not show more adverse pregnancy outcome compared with the controls, except for the higher cancellation rate as previously reported [36]. However, PCOS patients may still undertake a higher risk for abortion, and our failure to detect any statistical significance might be ascribed to the limited sample size. Second, the FF we analyzed could only reflect AA metabolism as a whole, but failed to correspond to each oocyte and its further developmental competence. In addition, we did not analyze whether the follicle size had any effect on AA metabolism. Future studies with a larger sample size and more intricate study design may help resolve these limitations.

## Conclusion

Both PCOS and obese patients exhibited metabolic disturbances of follicular AAs, which may exacerbate their IVF outcome possibly through changing glucose metabolism and/or inducing inflammation. These follicular AA metabolic disturbances may account for the higher abortion rate in PCOS patients and inferior pregnancy outcome in obese patients. Therefore, local metabolic disturbances should be taken into consideration when implementing diagnostic and therapeutic strategies. Moreover, treatments normalizing systemic and local AA metabolism in PCOS and obese patients may create a beneficial environment for oocyte development.

## Competing interests

The authors declare that they have no competing interests.

## Authors’ contributions

JQ, RL and PL participated in the study concept and design, analysis and interpretation of data, and critical revision of the manuscript. C-MZ did the statistical analyses and wrote the manuscript. YZ, YY and L-YY helped to collect the samples. N-NL and LL participated in sample preparation and laboratory assays. All authors are guarantors of the work. All authors participated in the revision and final approval of the manuscript, and had full access to the data of the study.

## Pre-publication history

The pre-publication history for this paper can be accessed here:

http://www.biomedcentral.com/1471-2393/14/11/prepub

## Supplementary Material

Additional file 1: Table S1 Follicular AA concentrations in PCOS and control subjects. **Table S2.** AA concentrations in patients with and without insulin resistance. **Table S3.** AA concentrations in pregnant and non-pregnant subjects. **Figure S1.** Pearson correlation analyis of follicular glycine and serine concentrations. Click here for file
