# Supplementary material for: Metabolic heterogeneity of follicular amino acids in polycystic ovary syndrome is affected by obesity and related to pregnancy outcome
Source: BMC Pregnancy Childbirth. 2014 Jan 10;14:11. doi: 10.1186/1471-2393-14-11 (PMC3897995; doi:10.1186/1471-2393-14-11)
Supplement: Additional file 1: Table S1 — Follicular AA concentrations in PCOS and control subjects. Table S2. AA concentrations in patients with and without insulin resistance. Table S3. AA concentrations in pregnant and non-pregnant subjects. Figure S1. Pearson correlation analyis of follicular glycine and serine concentrations. [file 1471-2393-14-11-S1.docx]

**Table S1 Follicular AA concentrations in PCOS and control subjects**

| **AAs** | PCOS (μM) | | | Control (μM) | | | *p*-value | | *p*-value | | *p*-value | | *p*-value | |
| --- | --- | --- | --- | --- | --- | --- | --- | --- | --- | --- | --- | --- | --- | --- |
|  | OPO (n=27) | | NOP (n=36) | OCO (n=11) | NOC (n=37) | OPO vs OCO | | NOP vs NOC | | OPO vs NOP | | OCO vs NOC | |  |
| ILE | 35.35±2.03 | 28.96±1.14 | | 32.18±3.13 | 28.15±1.28 | NS | | NS | | 0.005 | | NS | |  |
| LEU | 66.46±3.49 | 49.93±2.27 | | 57.73±4.50 | 51.17±3.15 | NS | | NS | | ＜0.001 | | NS | |  |
| VAL | 183.33±7.10 | 145.90±4.05 | | 159.00±10.73 | 141.24±5.19 | NS | | NS | | 0.001 | | NS | |  |
| LYS | 131.79±8.29 | 108.67±5.87 | | 124.83±11.11 | 109.57±4.51 | NS | | NS | | 0.022 | | NS | |  |
| MET | 15.68±1.25 | 13.46±0.89 | | 14.09±1.42 | 11.67±0.94 | NS | | NS | | NS | | NS | |  |
| PHE | 85.84±3.09 | 73.62±1.79 | | 76.16±4.01 | 67.43±1.81 | NS | | 0.018 | | ＜0.001 | | 0.033 | |  |
| THR | 66.26±4.76 | 56.76±2.74 | | 69.62±8.67 | 54.26±3.33 | NS | | NS | | NS | | NS | |  |
| HIS | 33.70±1.36 | 30.38±1.17 | | 33.62±2.88 | 31.51±1.70 | NS | | NS | | NS | | NS | |  |
| TRP | 36.68±1.55 | 34.74±1.24 | | 30.76±1.37 | 31.50±1.01 | 0.007 | | 0.045 | | NS | | NS | |  |
| ALA | 268.11±11.58 | 239.81±11.09 | | 280.82±32.25 | 268.08±14.37 | NS | | NS | | NS | | NS | |  |
| GLY | 109.23±8.69 | 119.86±6.75 | | 129.10±11.25 | 121.32±8.00 | NS | | NS | | NS | | N S | |  |
| SER | 42.78±2.32 | 39.83±2.02 | | 48.36±3.92 | 43.92±2.69 | NS | | NS | | NS | | NS | |  |
| ARG | 43.03±2.21 | 39.13±1.71 | | 47.16±5.31 | 35.62±1.40 | NS | | NS | | NS | | 0.004 | |  |
| PRO | 102.00±3.39 | 98.88±3.04 | | 115.18±5.78 | 107.10±4.84 | NS | | NS | | NS | | NS | |  |
| GLN | 285.15±17.53 | 271.75±13.12 | | 282.00±25.42 | 256.19±13.38 | NS | | NS | | NS | | NS | |  |
| GLU | 86.04±3.77 | 72.56±2.95 | | 86.30±7.56 | 63.27±2.56 | NS | | 0.020 | | 0.006 | | 0.001 | |  |
| ASN | 24.85±1.69 | 24.71±1.45 | | 22.82±2.58 | 21.38±1.86 | NS | | NS | | NS | | NS | |  |
| ASP | 3.69±0.37 | 3.20±0.35 | | 4.07±0.95 | 2.24±0.24 | NS | | 0.025 | | NS | | 0.009 | |  |
| TYR | 46.35±2.84 | 40.33±1.84 | | 38.67±2.36 | 34.44±1.53 | 0.045 | | 0.016 | | NS | | NS | |  |
| CYS | 68.97±3.97 | 60.79±2.81 | | 62.66±3.29 | 71.00±3.54 | NS | | 0.028 | | NS | | NS | |  |

Data are means ± SEM. Comparisons between groups were performed with t test after testing for equality of variance. OPO, overweight PCOS; NOP, normal-weight PCOS; OCO, overweight control; NOC, normal-weight control; NS, not significant.

**Table S2 AA concentrations in patients with and without insulin resistance**

| AAs | IR-PCOS (μM) | NIR- PCOS (μM) | IR- Control (μM) | NIR- Control (μM) | *p*-value |
| --- | --- | --- | --- | --- | --- |
|  | (n=26) | (n=37) | (n=4) | (n=44) |  |
| ILE | 33.82±2.15 | 30.21±1.21 | 28.63±4.98 | 29.11±1.28 | c |
| LEU | 63.85±3.93 | 52.21±2.32 | 52.93±10.60 | 52.65±2.77 | a,c |
| VAL | 176.27±7.48 | 151.89±4.91 | 142.50±12.31 | 145.56±5.11 | a,c |
| PHE | 83.20±3.27 | 75.81±1.98 | 66.25±2.16 | 69.72±1.88 | b,c |
| TRP | 34.79±1.56 | 36.12±1.24 | 30.00±4.66 | 31.45±0.83 | b |
| ALA | 279.54±11.70 | 232.54±10.21 | 333.75±7.31 | 265.30±14.04 | a |
| GLU | 86.18±3.46 | 72.82±3.15 | 58.63±6.68 | 69.45±3.13 | a,c |
| TYR | 44.12±3.00 | 42.06±1.86 | 35.40±1.36 | 35.40±1.36 | b,c |

Note: Data are means ± SEM.

a, statistically significant difference between IR-PCOS and NIR-PCOS (P<0.05).

b, statistically significant difference between NIR-PCOS and NIR-control (P<0.05).

c, statistically significant difference between IR-PCOS and NIR-control (P<0.05).

**Table S3 AA concentrations in pregnant and non-pregnant subjects**

| AAs | Group 1 ^a^ (μM) | | |  | Group 2 ^b^ (μM) | | |  | |
| --- | --- | --- | --- | --- | --- | --- | --- | --- | --- |
|  | PP (n=22) | | NPP (n=27) | *p*-value | PP(n=47) | NPP (n=47) | *p*-value | |  |
| BCAA | 235.65±12.02 | 263.47±12.78 | | 0.126 | 227.97±8.39 | 252.91±9.28 | 0.049 | |  |
| AAA | 157.40±5.99 | 162.34±6.04 | | 0.569 | 145.66±4.07 | 152.28±4.33 | 0.268 | |  |
| ILE | 29.43±1.85 | 33.47±1.91 | | 0.141 | 28.78±1.25 | 32.44±1.37 | 0.051 | |  |
| LEU | 53.54±3.61 | 59.27±3.63 | | 0.274 | 51.92±2.45 | 58.15±2.85 | 0.101 | |  |
| VAL | 152.68±7.05 | 170.74±7.85 | | 0.100 | 147.28±4.98 | 162.32±5.59 | 0.047 | |  |
| ALA | 267.32±13.34 | 251.67±14.12 | | 0.432 | 274.43±11.75 | 254.89±11.92 | 0.246 | |  |
| GLY | 132.73±9.56 | 110.78±7.01 | | 0.065 | 127.06±6.81 | 119.53±5.81 | 0.402 | |  |
| SER | 47.27±2.52 | 39.78±2.26 | | 0.032 | 46.40±1.96 | 43.04±2.01 | 0.234 | |  |
| ASP | 4.18±0..48 | 2.76±0.26 | | 0.009 | 3.14±0.33 | 3.00±0.27 | 0.749 | |  |

Data are means ± SEM. Comparisons between groups were done with t test after testing for equality of variance. PP, pregnant patients; NPP, non-pregnant patients.

a, PCOS group.

b, Patients considered as a whole.

**Figure S1 Pearson correlation analyis of follicular glycine and serine concentrations**

**
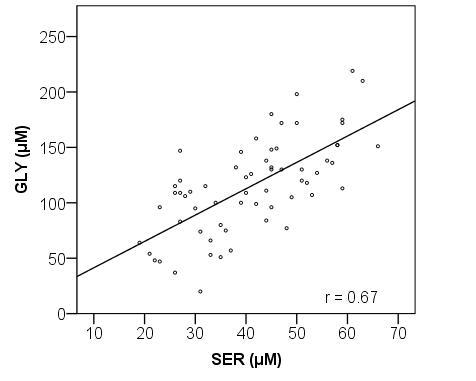
**
